# Tumor Budding in Colorectal Carcinoma—From Incidental Observation to Prognostic Marker: Lessons Learned from Colorectal Cancer Assessment

**DOI:** 10.3390/medicina62040734

**Published:** 2026-04-12

**Authors:** Aminia Diana Ciobănoiu, Valentin Titus Grigorean, Iancu Emil Pleşea, Răzvan Mihail Pleșea, Anwar Erchid

**Affiliations:** 1 Doctoral School, “Carol Davila” University of Medicine and Pharmacy, 050474 Bucharest, Romania; aminia-diana.ciobanoiu@rez.umfcd.ro (A.D.C.); anwar@yahoo.com (A.E.); 2 10th Clinical Department—General Surgery, Faculty of Medicine, “Carol Davila” University of Medicine and Pharmacy, 050474 Bucharest, Romania; 3 Department of Pathology, Bagdasar–Arseni Emergency Clinical Hospital, 041915 Bucharest, Romania; 4 Laboratory of Human Genomics, University of Medicine and Pharmacy of Craiova, 200349 Craiova, Romania; razvan_p1988@yahoo.com; 5 Regional Centre of Medical Genetics Dolj, Emergency County Hospital Craiova, 200642 Craiova, Romania

**Keywords:** tumor budding, prognostic marker, colon cancer

## Abstract

Tumor budding (TB), defined as isolated single cells or small clusters of up to four tumor cells at the invasive front of colorectal carcinoma (CRC), is recognized as an important histopathologic marker associated with adverse tumor behavior. This review summarizes current knowledge on the morphologic assessment, biological significance, and clinical relevance of TB, emphasizing emerging artificial intelligence (AI) methods that aim to automate and standardize its quantification. Standardized reporting by the International Tumor Budding Consensus Conference (ITBCC) has improved reproducibility, while novel deep-learning algorithms demonstrate potential for objective and prognostically relevant TB assessment. Integration of AI-based TB evaluation with molecular and stromal biomarkers may refine patient stratification and facilitate personalized treatment strategies.

## 1. Introduction

Tumor budding (TB) is a histologic manifestation of tumor cell dissociation and local invasion in CRC. First described more than six decades ago, TB has evolved into a recognized prognostic biomarker linked to lymphovascular invasion, lymph node metastasis, recurrence, and decreased overall survival [1,2,3,4]. The ITBCC (2016) established standardized definitions, grading, and reporting guidelines [5]. Beyond morphology, TB represents a morphologic correlate of epithelial–mesenchymal transition (EMT) and cell plasticity [6]. In parallel, advances in digital pathology and artificial intelligence (AI) have opened new possibilities for automated, reproducible assessment [7,8,9].

## 2. Methods—Selection Criteria

A comprehensive literature search was performed in the PubMed database for studies published between 2011 and the present. The search terms included ‘colorectal carcinoma’ and ‘tumor budding’. Additional relevant articles were identified through manual cross-referencing of the bibliographies of selected studies. Only articles published in English were included. Studies published prior to the International Tumor Budding Consensus Conference (ITBCC) were also included when relevant, particularly for comparison with the results and recommendations established by the consensus, as well as for providing general background information on colorectal cancer and tumor budding.

## 3. Morphologic Definition and Assessment

**Figure 1:**
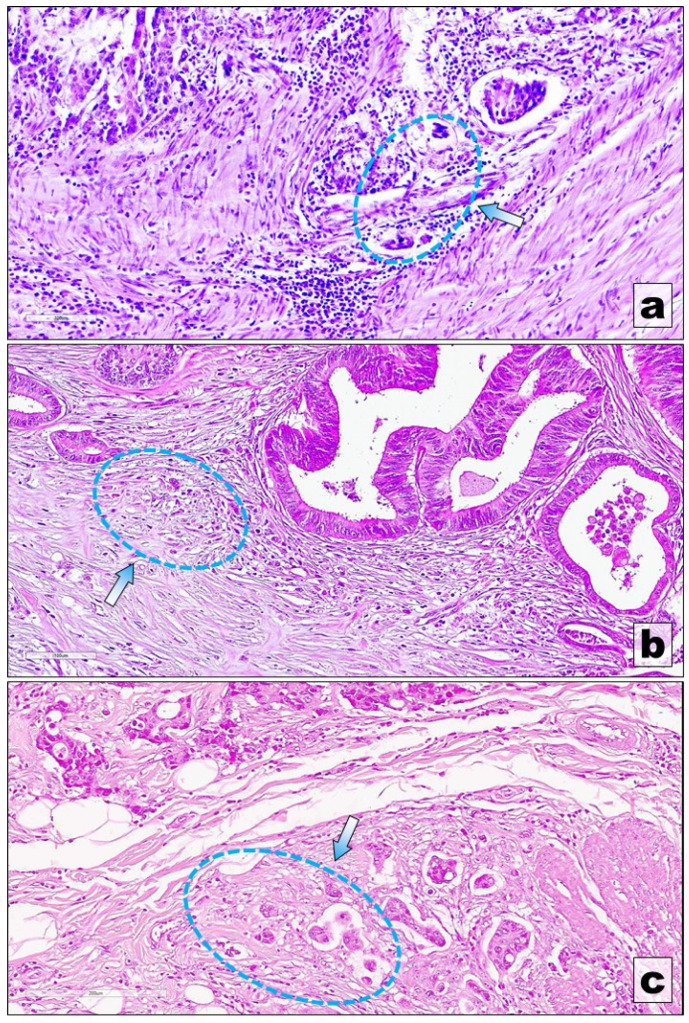
Budding Score in colorectal carcinoma (Grading system adapted from [3]). (**a**) Bd1; (**b**) Bd2; (**c**) Bd3; Illustrations from first author’s and corresponding author’s personal collection; Stain—Hematoxylin Eosin; Ob-X20—0.785 mm^2^ area (all pictures).

To harmonize the evaluation and reporting of tumor budding in colorectal cancer, the International Tumor Budding Consensus Conference (ITBCC) was convened, bringing together experts to establish uniform diagnostic criteria and assessment methods. The consensus defined tumor budding as the presence of single tumor cells or clusters composed of up to four tumor cells at the invasive front of colorectal carcinoma. Numerous studies have demonstrated that tumor budding represents a morphological manifestation of tumor cell dissociation and is strongly associated with aggressive tumor behavior, including lymph node metastasis and poor clinical outcomes. Based on the available evidence, the ITBCC recommends that tumor budding be assessed on routine hematoxylin and eosin-stained slides, as this approach is both cost-effective and globally applicable. Assessment should be performed by identifying a hotspot at the invasive front and counting tumor buds within a standardized field area of 0.785 mm^2^. To ensure standardization, tumor budding should be reported by area (mm^2^) rather than by objective lens magnification, as microscope field sizes vary. When the microscope field differs from 0.785 mm^2^, a conversion table should be used to normalize bud counts. For accurate hotspot selection, the invasive front should first be scanned in 10 separate fields at 20× magnification before counting buds in the single field showing the highest budding density. To facilitate clinical interpretation and improve prognostic stratification, a three-tier grading system was proposed, categorizing tumors into low (Bd1: 0–4 buds), intermediate (Bd2: 5–9 buds), and high budding (Bd3: ≥10 buds) (Figure 1).

In difficult cases, particularly in areas with marked stromal reaction or inflammation where small clusters of tumor cells may be difficult to distinguish from stromal cells on H&E-stained sections, pancytokeratin immunohistochemistry may be used as an adjunctive tool to facilitate the identification of tumor buds. By highlighting epithelial cells, it can improve the visualization of isolated tumor cells or small clusters. Nevertheless, pancytokeratin should be regarded only as a supportive tool and not as a mandatory component of routine tumor budding assessment, which remains based primarily on H&E evaluation [10,11]. Despite its recognized prognostic value in colorectal cancer, the assessment of tumor budding remains susceptible to interobserver variability. In a study by Hacking et al., which included 233 cases of colorectal carcinoma, interobserver reliability was evaluated among four pathologists using both the traditional two-tier grading system and the newer three-tier system proposed by the International Tumor Budding Consensus Conference (ITBCC). The results demonstrated only fair agreement, with agreement coefficients of 0.326 for the two-tier system and 0.25 for the ITBCC three-tier system. Notably, the older two-tier classification showed slightly better reproducibility than the more detailed three-tier grading system. The study also found that subspecialty training in gastrointestinal pathology was associated with improved observer agreement. These findings indicate that tumor budding assessment continues to be affected by reproducibility issues, underscoring the need for further standardization and the development of adjunctive methods to improve diagnostic consistency [12].

Despite its recognized prognostic value in colorectal cancer, the assessment of tumor budding remains susceptible to interobserver variability. In a study by Hacking et al., which included 233 cases of colorectal carcinoma, interobserver reliability was evaluated among four pathologists using both the traditional two-tier grading system and the newer three-tier system proposed by the International Tumor Budding Consensus Conference (ITBCC). The results demonstrated only fair agreement, with agreement coefficients of 0.326 for the two-tier system and 0.25 for the ITBCC three-tier system. Notably, the older two-tier classification showed slightly better reproducibility than the more detailed three-tier grading system. The study also found that subspecialty training in gastrointestinal pathology was associated with improved observer agreement. These findings indicate that tumor budding assessment continues to be affected by reproducibility issues, underscoring the need for further standardization and the development of adjunctive methods to improve diagnostic consistency [12].

## 4. Biological and Molecular Background

### 4.1. Tumor Budding and Epithelial–Mesenchymal Transition

Tumor budding in colorectal cancer is considered a morphological manifestation of tumor cell dissociation and acquisition of invasive properties. Several studies have suggested that tumor budding is closely associated with epithelial–mesenchymal transition (EMT), a biological process through which epithelial tumor cells lose their polarity and intercellular adhesion while acquiring migratory and invasive properties. This transition is thought to contribute to the detachment of single tumor cells or small clusters from the main tumor body, particularly at the invasive front.

At the molecular level, tumor budding has been linked to reduced expression of cell adhesion molecules, most notably E-cadherin, together with cytoskeletal reorganization. These changes facilitate the loss of epithelial cohesion and promote cellular dissociation, thereby favoring local invasion.

In addition, several signaling pathways involved in tumor progression have been implicated in the development of tumor budding. Among these, activation of the WNT/β-catenin pathway has been considered particularly relevant, as it contributes to increased cellular motility, invasiveness, and the acquisition of a more aggressive phenotype at the invasive front [13,14,15].

Beyond these intrinsic molecular alterations, the tumor microenvironment also appears to play an important role in the development of tumor budding. In particular, the local immune response at the invasive front has been shown to influence the budding phenotype. Several studies have demonstrated an inverse association between tumor budding and the density of tumor-infiltrating lymphocytes, especially cytotoxic T cells. Higher budding activity has been correlated with lower densities of intraepithelial CD3^+^CD8^+^ and CD3^+^CD8^+^CD45RO^+^ lymphocytes, suggesting that reduced local anti-tumor immune surveillance may facilitate tumor cell dissociation and invasion. These findings support the concept that tumor budding develops not only as a consequence of intrinsic molecular alterations within tumor cells, but also within a permissive and relatively immunosuppressed microenvironment [16].

### 4.2. Possible Role of Cancer Stem Cell-like Properties in Tumor Budding

Another proposed mechanism involved in the biology of tumor budding is the possible contribution of cancer stem cell (CSC)-like properties. The cancer stem cell concept is based on the idea that tumor progression may be driven, at least in part, by a subpopulation of undifferentiated tumor cells with enhanced capacities for invasion, resistance to apoptosis, and metastatic dissemination. In colorectal cancer, several studies have suggested a potential link between tumor budding, epithelial–mesenchymal transition, and stem cell-like behavior. This has led to the hypothesis that at least a subset of tumor buds may represent cells with CSC-like characteristics. Although current evidence remains heterogeneous and does not support a uniform stem cell phenotype in all tumor buds, these findings suggest that stemness-related mechanisms may contribute to the budding phenomenon [14].

### 4.3. Genetic and Molecular Features Associated with Tumor Budding

Building on the previously described interactions between tumor buds and the tumor microenvironment, tumor budding in colorectal cancer has also been associated with specific molecular alterations that contribute to its development. Several studies have demonstrated that tumor budding is linked to distinct genetic and molecular profiles that influence tumor cell behavior at the invasive front.

In particular, *KRAS* mutations have been associated with higher grades of tumor budding, suggesting that activation of the *RAS* signaling pathway may promote tumor cell dissociation and invasion. In contrast, microsatellite instability (MSI) has been reported to show an inverse relationship with tumor budding, with MSI-high tumors generally exhibiting lower budding activity, likely reflecting their strong immune microenvironment and increased lymphocytic infiltration. These findings further support the close interplay between tumor genetics and the tumor microenvironment in shaping the budding phenotype [17,18].

## 5. Clinical and Prognostic Significance

The clinical relevance of tumor budding in colorectal cancer lies not only in its association with adverse pathological features, but also in its utility across different clinical contexts. The retrospective International Budding Consortium (IBC) study evaluated the role of the International Tumor Budding Consensus Conference (ITBCC) score in predicting lymph node metastasis (LNM) and recurrence in an international cohort of 565 patients with pT1 colorectal cancer. The study focused on early invasive colorectal carcinomas, a clinically important subgroup in which treatment decisions following endoscopic or local excision rely heavily on the estimated risk of residual disease and nodal involvement.

The authors demonstrated that higher tumor budding grades were significantly associated with an increased risk of lymph node metastasis and recurrence, supporting the clinical value of tumor budding as a risk stratification marker in pT1 lesions. In this setting, tumor budding should be regarded not merely as a histologic indicator of tumor aggressiveness, but as a practical biomarker with direct therapeutic relevance, particularly in determining whether additional surgical resection may be warranted after local excision.

A major strength of the study lies in its support for the clinical applicability of the ITBCC scoring system in early colorectal cancer, where histopathologic risk assessment plays a pivotal role in patient management. The findings further support the inclusion of tumor budding among the key adverse histopathologic parameters considered in pT1 colorectal carcinoma, alongside lymphovascular invasion, poor differentiation, and depth of submucosal invasion.

Overall, the study highlights the particular relevance of tumor budding in pT1 colorectal cancer, as it may help identify patients at increased risk of nodal involvement or recurrence despite superficially invasive disease. This makes tumor budding especially valuable in post-polypectomy and post-endoscopic resection decision-making [19,20].

In stage II colorectal carcinoma, tumor budding is also of particular clinical importance, as its main utility lies in postoperative prognostic stratification following curative surgical resection. This is especially relevant in a subgroup of patients in whom conventional staging alone may not fully reflect biological behavior. In this context, the prospective multicenter study by Ueno et al., conducted within the SACURA trial, evaluated the prognostic and potential predictive significance of tumor budding in a cohort of 991 patients with stage II colon cancer.

The authors demonstrated that high-grade tumor budding was significantly associated with worse disease-free and overall survival, confirming its value as an independent prognostic factor in stage II disease. Importantly, tumor budding retained its significance even after adjustment for other clinicopathologic variables, reinforcing its role as a robust histopathologic biomarker.

The study also explored the relationship between tumor budding and adjuvant chemotherapy. Although adjuvant treatment with tegafur–uracil (UFT) appeared to be associated with an approximately 5% improvement in 5-year recurrence rates in BD2 and BD3 tumors, these differences did not reach statistical significance. Therefore, while the findings suggest that patients with higher budding counts may derive benefit from adjuvant therapy, they do not support a definitive predictive role for tumor budding in chemotherapy response. Rather, the main clinical relevance of tumor budding in stage II colorectal carcinoma remains its value in identifying patients at increased risk of recurrence following surgical resection [21] (Table 1).

Recent advances have further refined the understanding of tumor budding by placing it within a broader spectrum of infiltrative tumor growth patterns at the invasive front of colorectal cancer. In a large study integrating spatial transcriptomics, immunohistochemistry, and artificial intelligence-based analysis of over 1100 stage I–III colorectal cancer cases, tumor budding and poorly differentiated clusters were shown to represent a biological continuum of tumor cell dissociation rather than entirely separate entities. Tumor buds, defined as single cells or small clusters, exhibited the highest invasive potential compared with larger clusters, supporting their role as the most aggressive form within this spectrum. The study also demonstrated that smaller clusters, particularly two-cell units, are the most frequent structures at the invasive margin. Importantly, the combined assessment of tumor budding and poorly differentiated clusters provided superior prognostic performance compared with either parameter alone. These findings suggest that tumor invasion should be viewed as a dynamic and continuous process characterized by progressive reduction in cluster size, and highlight the potential of integrating multiple morphological parameters to improve risk stratification in colorectal cancer [22].

Koelzer et al. investigated the use of pancytokeratin immunohistochemistry for tumor budding assessment in colorectal cancer in a cohort of 386 cases. Tumor budding was evaluated using AE1/AE3 immunostaining, and the authors showed that this method could be prospectively implemented in routine diagnostic practice. In the subgroup of stage II colorectal carcinomas, immunohistochemically assessed tumor budding retained prognostic significance. These findings suggest that pancytokeratin-based assessment may be a feasible option in larger or specialized centers, where technical resources and workflow allow its application, although H&E remains the standard method for routine evaluation [23] (Table 1).

**Table 1:** Selected studies evaluating tumor budding as a prognostic factor in colorectal carcinoma.

Author (Year)	Study Design	Cohort (*n*)	Assessment Method	Main Finding	Ref.
Lugli A (2017)				ITBCC validation; prognostic significance across stages	[5]
Ueno H (2006–2010) Sacura Trial	Prospective multicenter study	991 patients with stage II colorectal carcinoma	H&E, one hotspot at invasive front, 0.785 mm^2^, ITBCC 3-tier system	High-grade tumor budding was independently associated with worse disease-free and overall survival	[21]
Hacking S (2019)	Retrospective study	233 patients	H&E, ITBCC scoring, hotspot method, 2-tier vs. 3-tier grading (multi-observer)	Tumor budding assessment showed only fair interobserver agreement; subspecialty training improved consistency	[12]
Koelzer VH (2017)	Prospective study (cohort 1) Retrospective study (cohort 2)	386 patients with stage II colorectal carcinoma (236 cases in cohort 1 and 150 cases in cohort 2)	AE1/AE3 pancytokeratin IHC, 10 HPFs (0.238 mm^2^), low/high-grade cut-off at 10 buds	CK vs. H&E evaluation: CK detects more buds, moderate interobserver agreement; useful adjunct to standard scoring	[23]

ITBCC: International Tumor Budding Consensus Conference.

## 6. Artificial Intelligence in Tumor Budding Assessment

Manual TB evaluation is labor-intensive and subject to inter-observer variation. To address these limitations, recent studies have explored automated and data-efficient deep learning models capable of detecting and segmenting tumor buds on histological images, even in settings with limited annotated data. These approaches aim to standardize tumor budding quantification, improve reproducibility, and reduce the burden on pathologists. At the same time, they open new perspectives for large-scale analysis and integration with other morphological and microenvironmental parameters. Together, these advances mark a transition from purely descriptive morphology toward a more quantitative and computationally driven framework, positioning tumor budding as a central element in the development of next-generation diagnostic and prognostic tools in colorectal cancer [24,25,26,27].

Recent advances in digital pathology have enabled the development of artificial intelligence (AI)-based approaches for histopathological analysis on a large scale. In the study by Campanella et al., a weakly supervised deep learning framework was applied to an exceptionally large dataset of 44,732 whole-slide images from 15,187 patients, without the need for detailed manual annotations. The algorithm demonstrated excellent diagnostic performance, achieving area under the curve (AUC) values above 0.98 across multiple tumor types. Importantly, the model was able to prioritize slides according to tumor probability, allowing pathologists to exclude approximately 65–75% of slides while maintaining 100% sensitivity at the case level. Although not specifically designed for tumor budding assessment, this study provides strong proof of concept that AI can process whole-slide images at scale, reduce workload, and improve efficiency in histopathological evaluation. These findings are highly relevant for tumor budding analysis, where manual hotspot selection and bud counting are time-consuming and subject to interobserver variability, suggesting that AI-based tools could significantly enhance reproducibility and standardization in routine practice [24]. Another CNN-based approach was developed to automatically identify tumor buds on hematoxylin and eosin-stained slides, addressing the well-known limitations of manual evaluation, including time consumption and interobserver variability. The dataset consisted of digitized colorectal cancer slides, with an average of approximately 115 tumor buds per slide, providing a substantial number of annotated budding structures for model training. The proposed method employed a weakly supervised learning strategy, allowing the algorithm to learn from partially annotated regions rather than requiring exhaustive manual labeling. In validation using cross-validation techniques, the model achieved high performance, with a precision of approximately 0.94 and a recall of 0.86, indicating reliable detection of tumor buds. Importantly, this approach demonstrated improved generalizability and stability compared with traditional fully supervised methods, particularly in the presence of noisy or heterogeneous annotations. These findings highlight the potential of AI-based systems to standardize tumor budding assessment, reduce observer-dependent variability, and facilitate large-scale, reproducible evaluation in routine pathology practice [28] (Table 2).

In their 2023 study, Bokhorst et al. developed a deep learning algorithm using a semi-supervised approach to detect tumor buds on pancytokeratin-stained whole-slide images, addressing the limitation of small annotated datasets. The model demonstrated a sensitivity of approximately 91% and showed a strong correlation with human expert annotations, confirming the reliability of automated bud detection. Importantly, the study showed that AI-derived tumor budding counts achieved a prognostic value comparable to manual assessment, supporting their clinical applicability. In addition to conventional bud counting, the authors proposed novel quantitative parameters, such as tumor bud density and spatial dispersion, which may provide additional prognostic information beyond the standard ITBCC scoring system. These findings suggest that AI-based tumor budding assessment can improve reproducibility and efficiency while maintaining clinical relevance, and may serve as a complementary tool to traditional histopathological evaluation in routine practice [25,29] (Table 2). This demonstrates not only the feasibility of automated TB scoring but also its potential to improve reproducibility and reduce workload in pathology workflows. Ultimately, incorporating such pipelines into routine diagnostics may enable more objective reporting of budding and facilitate large-scale outcome studies.

Such systems can standardize hotspot selection, quantify budding densities, and integrate stromal and immune features simultaneously.

In a systematic review by Lobanova et al., studies evaluating machine learning and deep learning approaches for the detection and prediction of tumor budding on histopathological images were analyzed. The authors reported that AI-based models demonstrate high diagnostic performance, with maximum accuracy values reaching up to 97.7% for tumor budding detection. Importantly, these approaches allow simultaneous evaluation of tumor budding and tumor microenvironment parameters, which are both critical for prognostic assessment. The review highlighted that AI systems can effectively process histological images and provide reliable quantification of tumor budding, potentially reducing subjectivity and interobserver variability associated with manual evaluation. Furthermore, AI models were shown to achieve high sensitivity and specificity across different studies, supporting their role as diagnostic support tools. However, the authors also emphasized limitations, including heterogeneity in performance metrics and relatively small datasets in some studies. Overall, these findings suggest that AI-based platforms have the potential to serve as adjunctive tools in routine pathology practice, improving the accuracy, reproducibility, and efficiency of tumor budding assessment in colorectal cancer [9]. Incorporating tumor budding into routine reporting could therefore improve risk stratification within stage III patients, allowing for a more nuanced assessment beyond conventional staging parameters. This may have direct clinical implications, particularly in guiding adjuvant treatment decisions, as patients with high tumor budding may benefit from more intensive therapeutic approaches or closer surveillance. Ultimately, the integration of tumor budding into standard pathological evaluation supports a more personalized approach to colorectal cancer management, bridging the gap between morphological assessment and clinical decision-making [30].

## 7. Limitations of AI-Based Tumor Budding Assessment

Despite the growing interest in artificial intelligence-based approaches for tumor budding assessment, several important limitations continue to restrict their translation into routine diagnostic practice. One of the main challenges is the lack of robust external validation, as many currently available models have been developed and tested within relatively controlled, single-center settings. This raises concerns regarding their reproducibility and generalizability across different institutions, where variations in scanners, image quality, tissue processing, and staining protocols may significantly affect performance.

Another important limitation relates to the inherent variability in the tumor budding assessment itself. Because manual bud identification is subject to interobserver variation, the annotations used to train AI models may also lack full consistency. In addition, differences in staining methods and in the histologic criteria used to define tumor buds may further complicate model development and validation. These issues are particularly relevant when comparing H&E-based and pancytokeratin-based approaches, which may not always generate equivalent reference standards.

Taken together, these methodological and translational challenges suggest that, although AI-assisted tumor budding assessment is a promising field, its current role remains primarily supportive. Further standardization, multicenter validation, and integration with routine pathological workflows are needed before such tools can be reliably implemented in everyday clinical practice [28,31].

## 8. Future Perspectives and Conclusions

Tumor budding (TB) has established itself as a key histopathologic biomarker in colorectal cancer, reflecting tumor invasiveness and providing clinically relevant prognostic information beyond conventional staging systems. Its incorporation into routine pathological reporting represents a significant step toward bridging traditional morphological assessment with tumor biology. Nevertheless, challenges remain, particularly regarding interobserver variability and the subjective nature of manual evaluation. In this context, the integration of artificial intelligence-assisted quantification offers a promising avenue to enhance reproducibility, standardization, and efficiency, while enabling large-scale, objective analysis of tumor budding. Moving forward, further efforts are required to develop explainable and clinically interpretable AI models, alongside robust multicenter validation to ensure generalizability across diverse clinical settings. Additionally, the combined assessment of tumor budding with immune and molecular parameters holds considerable potential for refining prognostic stratification and guiding personalized therapeutic approaches. Altogether, tumor budding exemplifies the convergence of conventional histopathology and emerging computational technologies, underscoring a paradigm shift toward precision diagnostics in colorectal cancer.

## Figures and Tables

**Table 2:** Representative AI-based methods for automated tumor budding quantification.

Study	Methodology	Performance	Ref.
Campanella et al. (2019)	AI-based weakly supervised CNN, H&E whole-slide images (*n* = 15,817 CRCs)	Good bud detection on H&E with reduced need for manual annotation	[24]
Bokhorst J.M. (2023)	Fully automated CNN pipeline on H&E whole-slide images (*n* = 981 CRCs)	AI-derived tumor bud density strongly correlated with manual ITBCC scores; independent prognostic biomarker for survival; robustness across scanners validated	[25]
Sajjad U. (2024)	Weakly supervised deep-learning framework (Bayesian Multiple Instance Learning) on H&E slides (*n* = 99 CRCs)	Achieved precision 0.95–0.97 for tumor-bud detection; demonstrated high accuracy without IHC; supports integration into digital pathology workflows	[28]

CNN: convolutional neural networks; ITBCC: International Tumor Budding Consensus Conference; CRC: Colorectal Carcinoma; IHC: Immunohistochemistry.

## Data Availability

Data are contained within the article.
